# Intestinal Effects of Brewers’ Spent Grain Extract In Ovo (*Gallus gallus*)—A Pilot Study

**DOI:** 10.3390/ani15030303

**Published:** 2025-01-22

**Authors:** Melissa Y. Huang, Louisa M. Smieska, Elad Tako

**Affiliations:** 1 Department of Food Science, Cornell University, Ithaca, NY 14853, USA; mh2284@cornell.edu; 2 Cornell High Energy Synchrotron Source, Cornell University, Ithaca, NY 14853, USA; lmb327@cornell.edu

**Keywords:** broiler chicken (*Gallus gallus*), intra-amniotic administration (in ovo), synchrotron X-ray fluorescence microscopy (µXRF), duodenal morphology, cecal microbiota, tannins, upcycling

## Abstract

Upcycling brewers’ spent grain (BSG) into poultry feed needs to be optimized. Since broiler chickens inefficiently digest fiber, we created a water-soluble BSG extract (BSGE) to explore this fraction’s potential nutritional benefits. We utilized intra-amniotic administration (in ovo) to target the gastrointestinal tract of broiler embryos. BSGE increased villus surface area and goblet cell quantity and size, implying improved duodenal development. The extract also changed cecal *Escherichia coli* (*E. coli*) and *Clostridium* abundances. Synchrotron X-ray fluorescence microscopy, along with zinc and iron transporter relative expression, did not reveal significant changes by BSGE. These findings highlight the potential for BSGE to be a functional feed component, underscoring the potential value of upcycling this byproduct. This pilot study supports future work exploring the impact of BSGE within feed and its effects over long-term consumption.

## Simple Summary

Poultry farmers face the challenge of optimizing flock nutrition and growth while minimizing feed costs. Sustainable strategies are even more urgent in the wake of antimicrobial growth promoter restrictions and rising demands for poultry meat. An innovative approach is upcycling brewers’ spent grain—the major byproduct of beer production—into poultry feed. Brewers’ spent grain is readily available, with approximately 36.4 million tons produced annually. However, monogastric animals like broiler chickens cannot efficiently digest fiber-rich ingredients like brewers’ spent grain. We turned to the water-soluble fraction of brewers’ spent grain and evaluated the effect of a brewers’ spent grain extract on gut development and function. We observed changes in duodenal morphology and cecal microbiota which support further assessments of brewers’ spent grain to promote sustainable practices as well as broiler chicken gut health.

## 1. Introduction

Brewers’ spent grain (BSG) is the main byproduct of beer production and a prime candidate for upcycling [1,2,3]. BSG is the semi-solid fraction separated from wort (which ultimately becomes beer), mainly consists of grain husks and seed coats, and contains around 70% fiber and 20% protein [2,4]. BSG accounts for 85% of brewing waste, with approximately 36.4 million tons available worldwide and 10 million tons in the United States alone [5]. Occasionally, BSG is reincorporated into cattle feed; however, BSG accumulation overwhelms current upcycling efforts [1,5]. Thus, other production animals, like broiler chickens, are being considered to redirect BSG surplus from landfills.

Optimal broiler feed ingredients should be abundant, inexpensive, and highly digestible [6]. BSG is a low-opportunity-cost feed material; however, the high fiber content in BSG is largely indigestible for monogastric animals like broiler chickens [7,8]. Unlike ruminants, broiler chickens lack the ability to ferment their feed [8,9]. Ruminal microbial fermentation increases non-starch polysaccharides digestibility while providing a major source of volatile fatty acids, essential amino acids, and micronutrients [9,10]. Without this fermentative digestive function, broiler chickens require highly digestible, energy-rich, and micronutrient-supplemented diets [11]. Since greater than 20% feed incorporation of BSG compromises broiler weight gain and gut health, it is recommended to pretreat fiber-rich ingredients like BSG enzymatically, chemically, and or physically [1,4,7]. However, these pretreatments are often expensive to implement [6]. In this study, we processed BSG into a water-soluble BSG extract (BSGE) to investigate whether BSG without its dietary fiber and protein fractions can impart nutritional benefits in vivo.

BSG contains a variety of phenolic compounds which are well accepted for their antioxidative, immune-regulatory, and anti-microbial potential [4,12,13,14,15,16]. BSG tannins are of particular interest because they are water-soluble, and in vitro studies suggest they may confer significant improvements in nutrient absorption and even protection against bacterial pathogens if dosed appropriately [17,18,19,20]. We utilized intra-amniotic administration (in ovo), a targeted approach on the broiler gastrointestinal tract, to evaluate the potential impact of BSGE on the broiler gut [21,22,23,24,25]. This method allows us to isolate the intestinal impact of a chosen sample (BSGE, in this case), without considering a solid diet or other management and environmental conditions. We examined duodenal morphology, cecal microbial populations, and zinc and iron parameters. Specifically, we employed synchrotron X-ray fluorescence microscopy (µXRF) to visualize the localization and concentration of elemental zinc and iron across duodenal tissue as a potential novel technique for assessing mineral status. Synchrotron µXRF can quantify a broad range of element concentrations in a single measurement with minimal sample preparation, making it a powerful tool for nutrient quantification in biological tissues [26,27]. We paired this with measurements of ZnT1, ZIP5, ZIP14, and Ferroportin mRNA to show the potential impact of BSGE on zinc and iron regulation. Our aim with this pilot study was to provide in vivo data to support further long-term assessments of BSGE in broiler chicken feed.

## 2. Materials and Methods

### 2.1. Brewers’ Spent Grain Extract (BSGE) Preparation

Brewers’ spent grain extract (BSGE) was prepared as previously described to ensure compatibility with the in ovo model [28]. Briefly, dried barley brewers’ spent grain was obtained from Harpoon Brewery (Boston, MA, USA), heated in 18 MΩ H_2_O (Type 1 water) at 60 °C for 60 min (water bath), and centrifuged for 60 min at 830× *g* (Allegra X-30R Centrifuge, Beckman Coulter, Brea, CA, USA). The supernatant was diluted to 5% with 18 MΩ H_2_O to ensure the osmolarity was less than 600 mOsm [25]. This prevents dehydrating the broiler embryos [25]. This 5% solution will now be referred to as brewers’ spent grain extract (BSGE). BSGE was stored at −20 °C.

### 2.2. High-Performance Liquid Chromatography (HPLC) Analysis of BSG and BSGE

Samples were sent to E & J Gallo (Modesto, CA, USA) to conduct compositional analyses [29]. For whole BSG, 100 g dry BSG was added to 500 mL of 50% ethanol and adjusted to pH 2.0. The mixture was agitated for 12 h at 25 °C then centrifuged at 5770× *g* for 10 min (Avanti J-E Centrifuge, Beckman Coulter, Brea, CA, USA). The supernatant was filtered through a 0.45 µm cellulose acetate filter with a glass microfiber prefilter layer (Whatman Puradisc, Cytiva Life Sciences, Marlborough, MA, USA) and stored at 5 °C until further analysis. For BSGE, the supernatant from Section 2.1 (before diluting to 5%) was directly analyzed.

Acetonitrile (HPLC grade), phosphoric acid, and additional consumables were purchased from VWR International (Radnor, PA, USA). Deionized water was obtained by an ELGA LabWater Purelab Flex System (Woodridge, IL, USA). The following HPLC standards were purchased from Sigma-Aldrich (St. Louis, MO, USA): gallic acid (CAS 149-91-7, ≥97.5%), protocatechuic acid (CAS 99-50-3, ≥97%), syringic acid (CAS 530-57-4, ≥95%), caffeic acid (CAS 331-39-5, ≥98%), *p*-coumaric acid (CAS 501-98-4, ≥98%), ferulic acid (CAS 537-98-4, ≥99%), astilbin (CAS 29838-67-3, ≥98%), (+)-catechin hydrate (CAS 225937-10-0, ≥98%), (−)-epicatechin (CAS 490-46-0, ≥98%), and quercetin dihydrate (CAS 6151-25-3, ≥99%). Oenin chloride (7228-78-6, ≥95%) was purchased from Indofine Chemical Company (Hillsborough, NJ, USA).

This method was adapted from a previous study [30]. BSG and BSGE samples were centrifuged at max speed of 21,100× *g* for 10 min at 10 °C (Sorvall Legend Micro 21R Refrigerated Centrifuge, Thermo Scientific, Waltham, MA, USA) prior to analysis via HPLC coupled with a diode array detector (DAD). The system included an Agilent 1260 Infinity Micro Degasser (Model G1379B), High Performance Autosampler (Model G1367C), Thermostatted Column Compartment (Model G1316B), DAD (Model G1315C), a Zorbax C18 precolumn (2.1 × 12.5 mm, 1.8 mm) coupled to a Zorbax Eclipse C18 analytical column (2.1 × 100 mm, 1.8 mm) (Agilent Technologies, Santa Clara, CA, USA). Two mobile phases were used: phase A was water/phosphoric acid at 99.5:0.5 *v*/*v* and phase B was acetonitrile/phosphoric acid at 99.5:0.5 *v*/*v*. The flow rate was set to 0.2 mL/min and the column temperature to 40 °C. The binary gradient of the mobile phases started at 90%, then 81% at 30 min, 67% from 30.75–37.5 min, 5% at 40.5 min, then 90% from 43.5–46 min. The injection volume was 4 μL for each sample. Calibration curves were based on 6 points covering a range from 1.0 mg/L to 200 mg/L for all compounds of interest besides catechin with a range of 1.0 mg/L to 1000 mg/L. All compounds were quantified by their corresponding standards except hydroxycinnamic acid, which was expressed in caffeic acid equivalents, and polymeric tannins, expressed in catechin equivalents. After every twentieth sample, an additional continuous calibration verification (CCV) sample was spiked with a known concentration of (+)-catechin and quercetin. Recovery rates were calculated from the difference between unspiked and spiked samples divided by the spiked concentrations. Recovery rates were 97–103% for CCV samples.

### 2.3. Intra-Amniotic Administration (In Ovo)

A total of 36 fertile Cornish Cross broiler (*Gallus gallus*) eggs were purchased from a commercial hatchery (Moyer’s Chicks, Quakertown, PA, USA). Eggs were incubated under optimal conditions (37 °C, 30 °C wet bulb temperature) at the Cornell University Animal Science Poultry Farm (Ithaca, NY, USA) (Model I-UNIT, Serial Number: 3695-01E-3189, NatureForm Hatchery Systems, Jacksonville, FL, USA) [31]. All animal protocols were approved by the Cornell University Institutional Animal Care and Use Committee (IACUC #2020-0077) and adhered to for the duration of this study.

On day 17 of incubation, 25 viable eggs, confirmed via candling, were randomly divided so that all three treatment groups had an equal, normal distribution of egg weight [25]. The treatment groups were as follows: no injection (negative control) (*n* = 9), 18 MΩ H_2_O (sham control) (*n* = 9), and BSGE (*n* = 7). The injection site (amniotic fluid avoiding large blood vessels) was confirmed by candling and cleaned with 70% ethanol. Then, 1 mL of 18 MΩ H_2_O or BSGE was injected per egg with sterile, single-use 25-gauge needles (BD, Becton, Dickinson and Company, Franklin Lakes, NJ, USA) [21]. The injection site was immediately sealed with cellophane tape, and eggs were returned to a hatcher (37 °C, 30 °C wet bulb temperature), injection site facing up.

On day 21 of incubation (4 days after intra-amniotic administration), hatchlings were weighed then euthanized via carbon dioxide inhalation. Duodenal loops were immediately placed in 10% neutral buffered formalin (HT501128, Sigma Aldrich, St. Louis, MO, USA). Remaining duodena and complete ceca were collected per hatchling, immediately placed in liquid nitrogen, then transferred to −80 °C until further analyses.

### 2.4. Duodenal Histology

**Figure 1:**
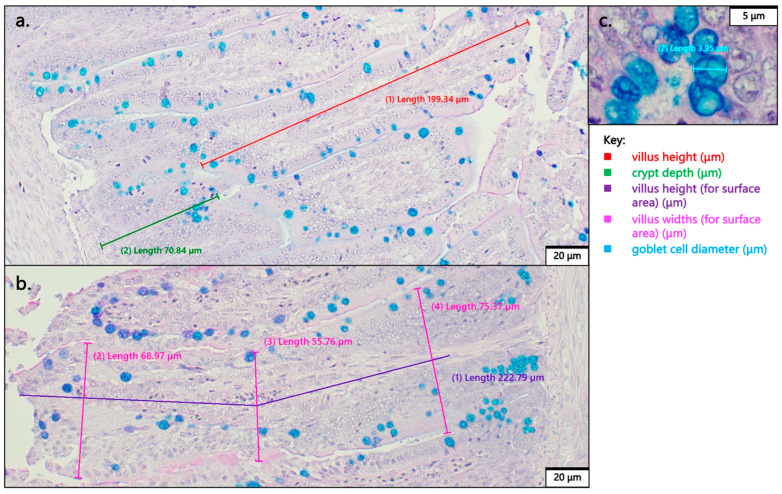
Example morphological measurements on duodenal tissue stained with Periodic Acid–Schiff and Alcian Blue from no injection control (Panel (**a**)), 18 MΩ H_2_O sham control (Panel (**b**)), and BSGE (Panel (**c**)). Panel a provides an example of villus height (red) to crypt depth (green) measurements. Panel (**b**) provides an example of villus surface area (widths = pink, height = purple) measurements. Panel (**c**) provides an example of goblet cell size (light blue) measurement. (Panels (**a**,**b**)) were captured at 20× magnification with 20 µm scale bars on the bottom right. Panel (**c**) was captured at 100× magnification with a 5 µm scale bar on the top right.

Samples were prepared as previously described [32,33,34]. Formalin-fixed duodenal loop samples were sectioned transversely to reveal a circular cross-section for villus analysis. Cross-sections were dehydrated, cleared, and embedded in paraffin wax. Serial 5 µm thick cross-sections were cut, placed on slides, deparaffinized in xylene, rehydrated in a graded alcohol series, and stained with Periodic Acid–Schiff and Alcian Blue. Slides were examined under an Olympus BX43 manual system microscope fitted with a DP22 2.8-megapixel digital camera (Olympus Life Science Solutions, Tokyo, Japan). cellSens Imaging Software Version 3.1 (Olympus Life Science Solutions, Tokyo, Japan) was used to view and analyze the microscope images. Morphometric measurements of villus surface area, goblet cell counts (per villi), goblet cell diameter, and crypt depth were collected. Villus surface area was calculated from the villus height and an average of three villus widths assuming the villi were perfect cylinders (Figure 1). Villus height was defined as the vertical distance from the villus tip to the villus–crypt junction or invagination (Figure 1). Goblet cells were distinguished based on their characteristic circular shape and purple to deep blue color, which corresponds to epithelial mucins (Figure 1).

### 2.5. Cecal Bacterial 16S rRNA PCR

As previously described, cecal content was homogenized with glass beads (4 mm diameter) (MP Biomedical, Santa Ana, CA, USA) and sterile 1X phosphate-buffered saline pH 7.4 (PBS) by vortexing (70× *g*) for 3 min (SCI-VS Variable Speed Vortex Mixer, Scilogex, Rocky Hill, CT, USA) [35]. The mixture was centrifuged at 1000× *g* for 5 min (Allegra X-30R Centrifuge, Beckman Coulter, Brea, CA, USA). The supernatant was collected and centrifuged at 4000× *g* for 10 min (Allegra X-30R Centrifuge, Beckman Coulter, Brea, CA, USA). The pellet was washed with 1X PBS and 50 mM ethylenediaminetetraacetic acid (EDTA) to extract DNA and then treated with 10 mg/mL lysozyme from chicken egg white (Sigma Aldrich, St. Louis, MO, USA) for 1 h at 37 °C. Bacterial DNA was isolated using a Wizard Genomic DNA Purification Kit (Promega Corporation, Madison, WI, USA) following the manufacturer’s protocol.

Primer sets for *Bifidobacterium*, *Lactobacillus*, *Clostridium*, *Escherichia coli*, and universal 16S rRNA were sourced from a previous study [35] and manufactured by Integrated DNA Technologies (IDT, Coralville, IA, USA) with standard desalting and nuclease-free water as the solvent. These microbial populations were chosen to represent the core broiler cecal microbiome at hatch with no exposure to a diet [36,37]. The PCR reaction followed the manufacturer’s recommendations: 12.5 µL GoTaq Green Master Mix 2X (Cat #M712, Promega Corporation, Madison, WI, USA), 2.5 µL upstream primer at 10 µM, 2.5 µL downstream primer at 10 µM, and 7.5 µL DNA template for a total reaction volume of 25 µL. The PCR reaction protocol was as follows: 94 °C for 3 min, 35 cycles of 94 °C for 30 s, 58 °C or 60 °C for 1 min, and 72 °C for 1 min, and finally, 72 °C for 5 min. Samples were stored at −20 °C until further analysis. PCR products were separated via electrophoresis on 2% agarose gels stained with ethidium bromide and quantified using the Image Lab Software (Version 6.0.1., Bio-Rad Laboratories, Hercules, CA, USA). Relative abundance was calculated as the ratio between the genera of interest ban intensity and the universal 16S rRNA band intensity.

### 2.6. Synchrotron X-Ray Fluorescence Imaging of Duodenal Tissue

Synchrotron X-ray fluorescence microscopy (µXRF) was performed at the Functional Materials Beamline (FMB) at the Cornell High-Energy Synchrotron Source (CHESS). An undulator source supplied X-rays to the FMB, and a beam energy of 9.7 keV was selected with a single side-bounce diamond monochromator and harmonic rejection mirror [38,39]. The X-ray beam was focused using a beryllium compound refractive lens (CRL) system (RxOptics GmbH & Co. KG, Monschau, Germany) to a spot size of 3 × 13 µm^2^ containing approximately 2 × 10^10^ photons/s. A 1-cm long ion chamber (AVS, Lansing, NY, USA) was placed downstream of the CRL to monitor incident intensity on the sample. XRF signal was measured using a Vortex ME4 detector (Hitachi, Japan) coupled with an Xspress3 digital signal processor (Quantum Detectors, Oxford, UK). XRF images were collected in fly-scanning mode, i.e., continuously moving the sample in the fast-scanning direction. Samples were first located in a coarse scanning mode (100 µm pixel sizes), and then fine scans were collected with 10 µm resolution and 100 ms/pixel dwell times. Typical scan times for a single map were on the order of 1–1.5 h. XRF signals were processed and fitted using the praxes software package (2022) developed at CHESS [40]. Image processing to address line artifacts was performed using custom Python code. Thin foil standards (Micromatter, Surrey, BC, Canada) were used to calculate the flux to concentration conversion and provide semi-quantitative concentration values in units of µg/cm^2^. Local concentration values were obtained from the tissue areas, excluding the surrounding paraffin, using a custom Python script with an interactive user-defined area selection tool.

### 2.7. Quantitative Reverse Transcription Polymerase Chain Reaction (RT-qPCR)

Duodenal tissue samples (30 mg) were collected, and total RNA was extracted with a RNeasy Mini Kit (Qiagen, Germantown, MD, USA) according to the manufacturer’s protocol. RNA was eluted in 50 µL of RNase-free water, then quantified with a NanoDrop 2000 (Thermo Fisher Scientific, Waltham, MA, USA), and assessed for quality by the absorbance ratio at 260/280 and 260/230 nanometers. Each RNA sample was diluted to 500 ng/µL with RNase-free water then stored at −20 °C.

cDNA was generated using a C1000 Touch Thermal Cycler (Bio-Rad Laboratories, Hercules, CA, USA) and an iScript cDNA Synthesis Kit (Bio-Rad Laboratories, Hercules, CA, USA) for a 20 µL reaction (4 µL reaction mix, 1 µL iScript reverse transcriptase, 1 µL sample RNA, 14 µL nuclease-free water). All reactions were performed according to the kit manual: 25 °C for 5 min, 46 °C for 20 min, 95 °C for 1 min, and 4 °C for holding. cDNA was stored at −20 °C.

ZnT1, ZIP5, ZIP14, Ferroportin, MUC2, and GAPDH primers were designed de novo with NCBI Primer-BLAST and manufactured by Integrated DNA Technologies (IDT, Coralville, IA, USA) with standard desalting. Primer sequences and accession numbers are shown in Table 1. The amplicon length was limited to 90–200 base pairs, primer lengths ranged from 17 to 22 base pairs, and the primers had >99% identity to the gene of interest via a BLAST search in the NCBI nucleotide database specific for *Gallus gallus* (taxid: 9031).

qPCR was performed in a CFX96 Touch (Bio-Rad Laboratories, Hercules, CA, USA) with 2 µL 2X SsoAdvanced Universal SYBR Green Supermix (Bio-Rad Laboratories, Hercules, CA, USA), 1 µL each of forward and reverse primers at 10 µM, 2 µL nuclease-free water, and 1 µL cDNA sample for a 10 µL reaction. Primer sets were optimized to efficiencies between 0.9 and 1.2. No template and no reaction controls were included per gene to ensure no contamination or unspecific amplification. qPCR was performed according to the manufacturer’s protocol: initial denaturation at 95 °C for 30 s, 40 cycles of denaturation at 95 °C for 15 s, annealing at 52–60 °C for 30 s, and elongation at 72 °C for 30 s. After cycling was complete, melt curves were generated from 65 °C to 95 °C, increasing every 5 s by an increment of 0.5 °C, to ensure the amplification of a single product. Relative gene expression was calculated via the Pfaffl method normalized to the expression of GAPDH and calibrated by an average of the no injection control group [41].

### 2.8. Statistical Analyses

This study followed a completely randomized design with independent observations. Initially, the data were checked for normality with the Shapiro–Wilk test. Homoscedasticity was measured via Bartlett’s test for normal data and Levene’s test for non-normal data at a significance level of 5%. Normal and homoscedastic data were analyzed with ANOVA followed by Tukey’s honestly significant difference (HSD) post hoc test. Non-normal and or heteroscedastic data were analyzed with Kruskal–Wallis tests followed by Dunn tests with Benjamini–Hochberg corrections [42]. Results presented in tables and figures were assessed at a significance level of 5%. Raw *p* values are included in text to allow readers to interpret the level of significance [43]. However, we use the word “significant” to describe data with a *p* value less than 0.05. All statistical analyses were performed using RStudio (v2023.06.2+561).

## 3. Results

### 3.1. Phenolic Composition of BSG and BSGE

Polymeric tannins were isolated in BSGE, while the other phenolic acids and flavonoids were not detected (Table 2). Notably, BSG contained 67.1 mg/L polymeric tannins, while BSGE contained 23.1 mg/L.

### 3.2. Duodenal Villi and Crypts

BSGE had the greatest villus surface area compared to both no injection (*p* = 2.953 × 10^−7^) and 18 MΩ H_2_O (*p* = 1.217 × 10^−9^) (Table 3). BSGE villi height was significantly greater than 18 MΩ H_2_O (*p* = 1.916 × 10^−5^) and unchanged compared to no injection (*p* = 0.258) (Table 3). BSGE crypt depth was significantly greater than no injection (*p* = 3.307 × 10^−8^), and significantly less than 18 MΩ H_2_O (*p* = 1.515 × 10^−18^) (Table 3). VH:CD was the greatest for no injection (*p* = 1.840 × 10^−6^) and least for 18 MΩ H_2_O (*p* = 1.859 × 10^−6^).

### 3.3. Duodenal Goblet Cells

BSGE had significantly more goblet cells per villus than no injection (*p* = 3.541 × 10^−7^) and 18 MΩ H_2_O (*p* = 1.550 × 10^−8^) and significantly larger goblet cells compared to no injection (*p* = 6.225 × 10^−6^) and 18 MΩ H_2_O (*p* = 1.069 × 10^−11^) (Table 4). However, BSGE had significantly fewer goblet cells per crypt compared to no injection (*p* = 1.494 × 10^−3^) and 18 MΩ H_2_O (*p* = 2.219 × 10^−7^) but significantly greater goblet cell size in the crypts compared to no injection (*p* = 2.311 × 10^−5^) and 18 MΩ H_2_O (*p* = 7.397 × 10^−4^) (Table 4).

### 3.4. Cecal Bacterial Abundances

**Figure 2:**
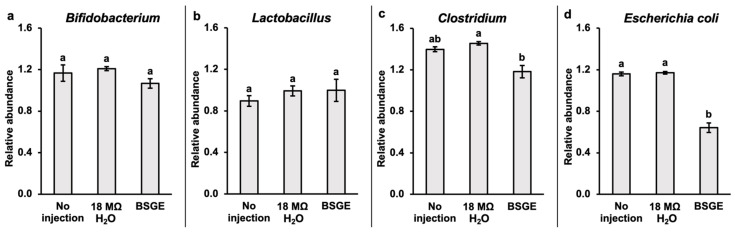
Values are presented as the relative abundance ± SEM, *n* = 5. ^a,b^ Treatment groups assigned different letter superscripts are significantly different (*p* < 0.05). Groups without letter superscripts had no significant differences. BSGE = brewers’ spent grain extract.

BSGE had lower relative abundance of *Clostridium* compared to 18 MΩ H_2_O (*p* = 0.00708) and no injection (*p* = 0.0990) (Figure 2c). BSGE also had lower relative abundance of *E. coli* compared to 18 MΩ H_2_O (*p* = 0.0140) and no injection (*p* = 0.0200) (Figure 2d). There were no differences in the relative abundances of *Bifidobacterium* (*p* = 0.1994) or *Lactobacillus* (*p* = 0.563) (Figure 2a,b).

### 3.5. Zinc and Iron Localization in Duodenal Tissue

**Figure 3:**
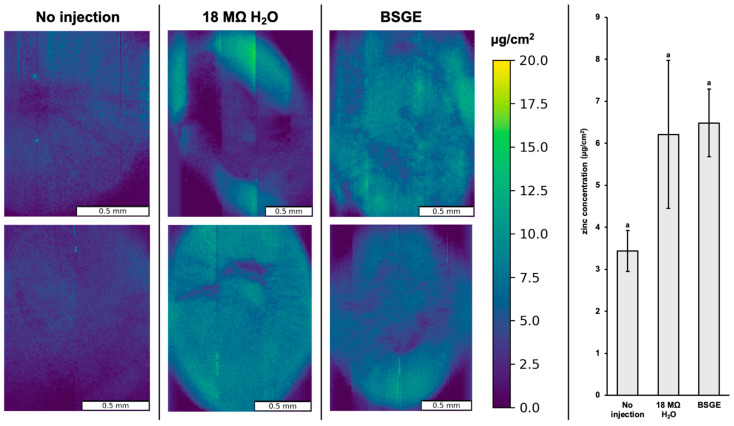
Zinc distribution scans of paraffin-embedded duodenal tissue for no injection, 18 MΩ H_2_O, and BSGE groups in biological duplicate. Average concentration of zinc (µg/cm^2^) across duodenal cross-sections with error bars representing the standard deviation. ^a^ Treatment groups assigned same letter superscript are not significantly different (*p* > 0.05). Note that all scans are plotted on the same concentration scale (µg/cm^2^) for direct comparison. Vertical streaks in the images are related to fluctuations in synchrotron current.

**Figure 4:**
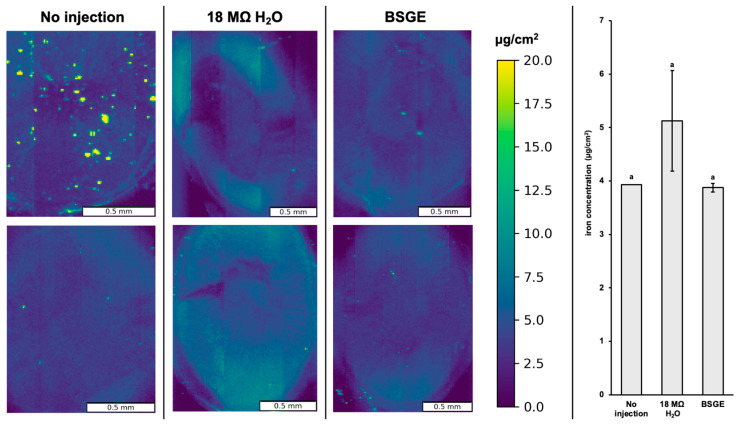
Iron distribution scans of paraffin-embedded duodenal tissue for no injection, 18 MΩ H_2_O, and BSGE groups in biological duplicate. The top leftmost scan was removed from concentration measurements due to debris. Average concentration of iron (µg/cm^2^) across duodenal cross-sections with error bars representing the standard deviation. ^a^ Treatment groups assigned same letter superscript are not significantly different (*p* > 0.05). Note that all scans are plotted on the same concentration scale (µg/cm^2^) for direct comparison. Vertical streaks in the images are related to fluctuations in synchrotron current.

There were no significant differences in zinc or iron localization and concentration across the three treatment groups (*p* = 0.265 and 0.497, respectively) (Figure 3 and Figure 4).

### 3.6. Relative Expression of Duodenal Proteins

**Figure 5:**
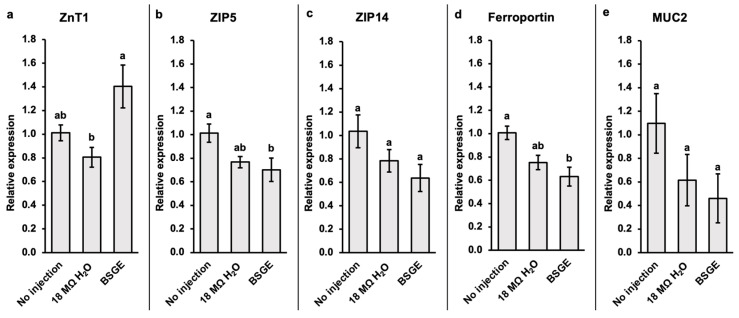
Values are presented as the mean fold change in expression relative to GAPDH (housekeeping gene). Error bars are the standard error of the mean (SEM), *n* = 5. ^a,b^ Treatment groups assigned different letter superscripts are significantly different (*p* < 0.05). BSGE = brewers’ spent grain extract; ZnT1 = zinc transporter 1; ZIP5 = zinc/iron-regulated transporter-like protein 5; ZIP14 = zinc/iron-regulated transporter-like protein 14; MUC2 = mucin 2 oligomeric mucus/gel-forming.

Data in Figure 5a–e are relative to the expression of GAPDH and calibrated by the no injection control group. ZnT1 was upregulated by BSGE compared to 18 MΩ H_2_O (*p* = 0.0267) and unchanged compared to no injection (*p* = 0.437) (Figure 5a). ZIP5 was downregulated by BSGE compared to no injection (*p* = 0.0385) and unchanged compared to 18 MΩ H_2_O (*p* = 0.828) (Figure 5b). ZIP14 expression did not significantly differ amongst the three groups (*p* = 0.0936) (Figure 5c). Ferroportin was downregulated by BSGE compared to no injection (*p* = 0.00533) and unchanged compared to 18 MΩ H_2_O (*p* = 0.444) (Figure 5d). There were no significant differences in MUC2 expression (*p* = 0.159) (Figure 5e).

## 4. Discussion

The current study assessed the impact of a brewers’ spent grain extract (BSGE) on duodenal morphology, cecal bacterial populations, zinc and iron localization and concentration, and gene expression of key proteins in ovo (*Gallus gallus*). The in ovo model allowed for a controlled investigation of the potential effects BSGE has on the broiler gut.

As expected, hydroxycinnamic acids and tannins were extracted from BSG (Table 2) [13,44]. The low quantities of ferulic acid and *p*-coumaric acid in BSGE could be due to the amount of hydroxyl groups present in these compounds, which is positively proportional to its solubility in ethanolic solvents [45]. The composition of BSGE was as expected due to the water extraction method employed. Solvents like ethanol and methanol are used due to their efficiency in extracting phenolic compounds; however, the in ovo model necessitates a water-based extract [45,46]. Thus, we only extracted significant quantities of polymeric tannins (Table 2) [47]. Future studies should consider varying the extraction procedure to gauge how the method and shifts in phenolic composition affect bioactivity in ovo.

### 4.1. Impact of BSGE on Duodenal Morphology

Histomorphology serves as a powerful visual indicator of gut development and health [48,49]. Small intestinal villi take on a finger-like shape to maximize their nutrient-absorptive surface. Thus, villus surface area is a measure of nutrient uptake capacity [48,49]. Villus surface area was the greatest for BSGE (*p* < 0.05) (Table 3), which could be due to a stimulating effect of tannins on intestinal enterocyte proliferation [19,50]. Significantly larger villi could indicate increased potential for nutrient uptake, attributable to the tannins in BSGE. However, the dose and type of tannins affect tissue differently [50,51]. Thus, this line of inquiry requires further study to determine if BSGE directly impacts cell proliferation pathways and enhances intestinal tissue growth in a dose-dependent manner.

Villus height-to-crypt depth ratio (VH:CD) is another morphological indicator of broiler intestinal health. A lower VH:CD value is associated with intestinal injury and higher turnover of the epithelial cells to repair the tissue [52,53]. VH:CD for BSGE was significantly greater than 18 MΩ H_2_O (*p* < 0.05) but significantly less than no injection (*p* < 0.05) (Table 3), suggesting BSGE did not significantly injure the duodenal lining. Future work should assess whether BSGE affects temporal shifts in villus turnover and development over long-term consumption.

A key measure of intestinal injury is the adequate production of mucus by goblet cells. A decrease in the size and abundance of duodenal goblet cells can indicate a reduction in mucin production and content, as well as an increase in the chances of intestinal injury [54,55,56]. Since BSGE had significantly more and larger villus goblet cells, as well as larger crypt goblet cells (*p* < 0.05) (Table 4), BSGE likely did not impair the tissue to require more mucus production [56,57,58]. We also observed no significant difference in the relative expression of MUC2 (Figure 5e), supporting the fact that no change in mucus production was required by the hatchlings after BSGE intra-amniotic administration. It is worth noting that the intestinal mucosal layer is especially important as a protective barrier, resulting in a rapid increase in MUC2 expression in the duodenum up until the day of hatch [59]. This upregulation in MUC2 could also have overshadowed smaller shifts in expression induced by BSGE. Since the in ovo model only encompasses the embryonic phase, future long-term studies in adult broilers should also assess MUC2 expression. This would help ascertain whether MUC2 is sensitive to developmental stages as a morphological biomarker of gut development.

### 4.2. Impact of BSGE on Cecal Bacterial Populations

Since the intestinal tract is constantly encountering external factors like food, other microbes, etc., the gut microbiota have a vital, dynamic role in maintaining tissue homeostasis [56,57,58,59]. Previous studies on tannins fed to adult broiler chickens also observed significant decreases in *E. coli* and *Clostridium* like the current study (Figure 2) [51,60,61]. The authors describe that polymeric tannins can form an insoluble layer of denatured proteins, covering the mucosal lumen of the intestines, as well as bacterial cell membranes [17,51,62]. This layer could protect and hinder bacterial colonization [51]. In addition, tannins have a higher affinity to iron than *E. coli*, which could explain the decrease in *E. coli* abundance as a result of potential nutrient competition [62]. Future work is needed to determine if the same decrease in *E. coli* and *Clostridium* abundance can be replicated over long-term consumption of BSGE within a diet. In addition, it would be important to investigate how the changes in bacterial flora from BSGE consumption affect physiological parameters such as tissue development and morphology.

### 4.3. Impact of BSGE on Zinc and Iron Localization, Concentration, and Transport in Duodena

Zinc and iron are essential to broiler growth, health, and welfare [63,64]. Additionally, gut microbiota compete with the host for these trace minerals, affecting energy acquisition and growth of both bacteria and the host [65]. Zinc and iron were of particular interest to describe the effects observed by BSGE in ovo.

We observed no significant differences in zinc and iron localization or concentration likely due to the low number of biological replicates (Figure 3 and Figure 4). In addition, XRF intensity is corrected for incident X-ray flux on the sample which can be imperfect when the total measured signal is low (i.e., tissue from broilers that had not consumed a solid diet, only intra-amniotic administration) (please see Supplementary Materials).

However, we observed certain changes in the regulation of certain zinc transporters. ZnT1 and ZIP5 are basolateral zinc exporters. ZnT1 is upregulated in zinc excess while ZIP5 is internalized and degraded in states of zinc excess [66,67,68]. ZIP14 is a manganese transporter and its upregulation is associated with zinc deficiency [69]. Since BSGE tended to upregulate ZnT1 and did not significantly change ZIP5 or ZIP14 expression (Figure 5a–c), it is possible that BSGE hatchlings were in a state of zinc excess. However, this cannot be confirmed without an assessment of physiological zinc status. Further optimization of paraffin-embedded tissue synchrotron imaging could provide a more sensitive visual indicator of zinc status to corroborate molecular analyses. For iron, ferroportin in duodenal enterocytes is more sensitive to systemic than cellular iron levels [70]. Since BSGE did not change ferroportin expression (Figure 5d) or iron localization and concentration (Figure 4), BSGE likely did not change systemic levels of iron. Our current findings require further study measuring zinc and iron in the lumen and circulation, as well as quantifying mineral transporter proteins to describe any mechanistic effects by BSGE.

### 4.4. An Elaboration on Synchrotron X-Ray Fluorescence Microscopy

Elemental analysis of fixed and paraffin-embedded tissues has not been widely employed in large part due to the unpredictable effects of chemical fixation on elemental redistribution. Frozen hydrated tissues are often regarded as the closest representative of in vivo elemental distribution, while chemical fixation or freeze drying have been demonstrated to result in significant leaching or preferential accumulation depending on the element and tissue type [71,72]. Therefore, with these caveats in mind, we discuss these results strictly as a comparison between identically treated samples, with the knowledge that the measured concentrations are likely not identical to in vivo statuses.

Another important caveat in interpreting this data is that µXRF alone is not a depth-specific measurement, and the sampled depth varies based on the energy of the elemental signal of interest. For the iron Ka line at 6.4 keV, the sampling depth in pure paraffin is estimated to be about 1–2 mm, while for the zinc Ka line at 8.6 keV, the sampling depth is on the order of 3–4 mm [73]. Therefore, the samples were mounted perpendicular to the X-ray microbeam, as opposed to the traditional 45-degree geometry, to avoid spatial blurring due to the sampling depth. Future studies would benefit from confocal µXRF, which is depth-specific and would likely result in improved spatial resolution within the tissues.

### 4.5. Study Limitations

We recognize the limitations of this study. Since this was a pilot study, the total sample size was limited to the 25 viable, fertile eggs for intra-amniotic administration. Future long-term analyses of BSGE with broilers should increase biological sample size. Experimental time at the Cornell High-Energy Synchrotron Source (CHESS) was limited so we were restricted to two biological replicates and two minerals for analysis. Despite these constraints, the chosen treatment groups were designed to ensure effective control of the BSGE treatment group. While our findings provide comparative zinc and iron concentrations in the duodenal tissue, further studies should include physiological status measurements to corroborate our µXRF scans and mRNA measurements. It would also be pertinent to measure the zinc and iron concentrations in BSGE to begin with. Thirdly, our bacterial population analyses would benefit from more powerful techniques such as 16S rRNA sequencing or shotgun metagenomics to better describe the cecal microbiota without bias. Despite these limitations, this pilot study initiates a necessary conversation around studying and utilizing BSGE in poultry feed to support intestinal health.

## 5. Conclusions

To successfully upcycle BSG in broiler feed, it is imperative to describe its potential impact on broiler gastrointestinal functionality. Due to the high fiber content in BSG, we isolated the water-soluble fraction of BSG (BSGE) to study its effects on the broiler intestinal tract. Our findings support conducting future long-term analyses of BSGE incorporated into broiler feed to not only increase the sustainability of poultry production but also improve the nutrition of broiler chickens. These future studies should investigate the dose-dependency of BSGE, employ mineral tracing, and conduct untargeted microbiome analyses and metabolomics to establish more mechanistic links between BSGE, microbial shifts, and gut function.

## Figures and Tables

**Table 1:** ZnT1, ZIP5, ZIP14, Ferroportin, MUC2, and GAPDH primer information.

Gene	Forward Primer (5′→3′)	Reverse Primer (5′→3′)	Amplicon Length (bp)	Annealing Temp (°C)	Accession #
ZnT1	AGCCAAGAGAAGCTGGGTGA	TTAAGCTGCGCGCTAGAGTC	119	52	NM_001389457.2
ZIP5	AGCTGATCTGGTGTGGATGG	GCGAGGTCACCCAGCTC	159	52	XM_025145573.3
ZIP14	GTTCTGCCCCGCTGTCCT	GGTCTGCCCTCCTCCGTCT	96	52	XM_040689606.2
Ferroportin	CTCAGCAATCACTGGCATCA	ACTGGGCAACTCCAGAAATAAG	98	60	NM_001012913.2
MUC2	CTCTGGCTGGCTCTTTCCAA	AATGGTTGTTCCCCCAGGTG	94	55	XM_421035.2
GAPDH	TCGGAGTCAACGGATTTGGC	TTCCCGTTCTCAGCCTTGAC	181	53	NM_204305.2

ZnT1 = zinc transporter 1; ZIP5 = zinc/iron-regulated transporter-like protein 5; ZIP14 = zinc/iron-regulated transporter-like protein 14; MUC2 = mucin 2 oligomeric mucus/gel forming; GAPDH = glyceraldehyde 3-phosphate dehydrogenase.

**Table 2:** Phenolic compounds in BSG and BSGE.

Compound	BSG (mg/L)	BSGE (mg/L)
	**phenolic acids**	
gallic acid	1.2	nd
protocatechuic acid	0.9	nd
syringic acid	nd	nd
*p*-coumaric acid	0.4	nd
ferulic acid	1.1	nd
other hydroxycinnamic acids	72.4	nd
	**flavonoids**	
astilbin	0.5	nd
catechin	nd	nd
epicatechin	32.0	nd
quercetin dihydrate	nd	nd
	**tannins**	
polymeric tannins	67.1	23.1

nd = not detected. BSG = brewers’ spent grain; BSGE = brewers’ spent grain extract.

**Table 3:** Villus surface area, height, and crypt depth.

	Villus Surface Area (µm^2^)	Villus Height (µm)	Crypt Depth (µm)	VH:CD
No injection	25,560.2 ± 715.5 ^b^	193.1 ± 3.8 ^a^	25.0 ± 1.0 ^c^	10.2 ± 0.5 ^a^
18 MΩ H_2_O	24,482.2 ± 697.1 ^c^	171.5 ± 4.0 ^b^	37.3 ± 1.1 ^a^	5.3 ± 0.2 ^c^
BSGE	33,286.7 ± 1048.8 ^a^	199.3 ± 4.9 ^a^	31.0 ± 0.6 ^b^	6.8 ± 0.2 ^b^

Values are presented as the mean ± SEM, *n* = 5. ^a–c^ Treatment groups assigned different letter superscripts significantly differ (*p* < 0.05) within the column. BSGE = brewers’ spent grain extract. VH:CD = villus height-to-crypt depth ratio.

**Table 4:** Goblet cell counts and sizes per villus and crypt.

	Villi		Crypt	
	Quantity	Diameter (µm)	Quantity	Diameter (µm)
No injection	12.6 ± 0.4 ^b^	3.37 ± 0.06 ^b^	6.4 ± 0.3 ^b^	2.92 ± 0.06 ^c^
18 MΩ H_2_O	11.9 ± 0.3 ^c^	3.15 ± 0.07 ^c^	7.1 ± 0.3 ^a^	3.04 ± 0.07 ^b^
BSGE	16.0 ± 0.5 ^a^	3.91 ± 0.07 ^a^	5.2 ± 0.2 ^c^	3.32 ± 0.06 ^a^

Values are presented as the mean ± SEM, *n* = 5. ^a–c^ Treatment groups assigned different letter superscripts significantly differ (*p* < 0.05) within the column. BSGE = brewers’ spent grain extract.

## Data Availability

The original contributions presented in this study are included in the article/supplementary material. Further inquiries can be directed to the corresponding author.

## Supplementary Materials

The following supporting information can be downloaded at: https://www.mdpi.com/article/10.3390/ani15030303/s1, Table S1: µXRF sample matrix reference values. Refs. [74,75] are cited in Supplementary Materials.
