# Supplementary material for: Intestinal Effects of Brewers’ Spent Grain Extract In Ovo (Gallus gallus)—A Pilot Study
Source: Animals (Basel). 2025 Jan 22;15(3):303. doi: 10.3390/ani15030303 (PMC11816252; doi:10.3390/ani15030303)
Supplement: Supplementary file 1 [file animals-15-00303-s001.zip › animals-3410115-supplementary.pdf]

Supplementary Materials

Table S1: μXRF sample matrix reference values.

Element	% mass / % atoms in protein	% mass / % atoms in fat	% mass / % atoms in water	% mass in broiler small intestine	% atoms in broiler small intestine
**Oxygen**	21.5 / 24	11 / 11	89 / 67	76.1	30.6
**Carbon**	52.5 / 12	77 / 19		7.6	1.8
**Hydrogen**	6.5 / 63	11.5 / 70	11 / 33	10.2	63.8
**Nitrogen**	16.5 / 1			1.9	0.1

Element maps were generated by XRF spectrum fitting in praxes, which employs PyMCA libraries [74]. The sample matrix was modeled as primarily oxygen, carbon, and nitrogen based on the table below – average composition chicken intestines [75], with a density of 1.02 g/cm^3 and thickness of 4 mm. The μXRF data was corrected for incident flux, but some dependence on the synchrotron current persisted in the fitted maps. To reduce the visual impact of these vertical artifacts in the μXRF maps, post-scan image processing was performed in Jupyter with custom Python scripts. First, an upper threshold (20 μg/cm^2) was applied to reduce the impact of any hot pixels. Next, the images were cropped closely to the tissue region of interest. In one case, a missing column of data was replaced with the mean of its neighbors. To level the image between synchrotron fills, we calculated a correction value for each column by scaling the image to a maximum value of 1, smoothing along the vertical axis, and taking the mean of each column, effectively capturing the synchrotron fill pattern. The image was then divided by this scaling factor column by column. This procedure reduced the tilts between synchrotron fills but does have the effect of amplifying noise at lower concentrations.
